# Optimal Mechanical Stretch Promotes TSP‐1 Expression Through Akt and GSK‐3β/β‐Catenin Signaling Pathways in Keloid Formation

**DOI:** 10.1002/hsr2.71275

**Published:** 2025-09-18

**Authors:** Xiangwen Xu, Yihan Zhang, Yanting Ou, Yixing Kang, Mengfan Wu, Jun Feng, Yun Long, Yongyan Cui, Dandan Liu, Lin Luo

**Affiliations:** ^1^ Department of Plastic and Reconstructive Surgery Peking University Shenzhen Hospital Shenzhen Guangdong China; ^2^ Department of Plastic and Reconstructive Surgery Shenzhen Xinhua Hospital Shenzhen China

**Keywords:** Akt, biomedical device, cell stretching, keloid, TSP‐1

## Abstract

**Background and Aims:**

The formation of keloids is influenced by mechanical stretch. Thrombospondin‐1 (TSP‐1) is identified as a tension‐sensitive protein. However, the relationship between TSP‐1 and keloid formation induced by mechanical stretch remains unknown.

**Methods:**

A simple customized mechanical stretch device was used for the application of homogeneous equibiaxial stretch (HES). Using Western blot and RT‐PCR, the optimal stretch strength and duration were determined. Regarding the functional changes induced by stretch in keloid fibroblasts (Kfbs), cell function assays were used. The relationship between TSP‐1 expression and stretch‐induced scar formation in human and animal models was investigated using immunohistochemistry. The knockdown of TSP‐1 in fibroblasts served as a reverse test.

**Results:**

Optimal HES (oHES) could be achieved with four rotations of the screws on our mechanical stretch device, resulting in a significant increase in vimentin, Col I, and fibronectin expression in Kfbs on Day 5. Additionally, oHES significantly promoted cell proliferation and migration. oHES resulted in the upregulation of TSP‐1 expression in both in vitro and in vivo experimental settings. The inhibition of TSP‐1 may attenuate oHES‐induced keloid formation through the Akt and GSK‐3β/β‐catenin signaling pathways.

**Conclusion:**

The results confirmed that oHES promoted keloid formation by increasing Col I expression through TSP‐1‐mediated Akt and GSK‐3β/β‐catenin signaling pathways.

## 1 Introduction

The prevalence of pathological scars resulting from car accidents and traumatic events is estimated to be as high as one million cases, and this number has been observed to rise over time. Severe pathological scarring, especially keloid, not only affects the patients' appearance and joints function, but also their quality of life [1]. Currently, keloids can be treated with glucocorticoid injection, surgical excision, radiation therapy, and laser‐based therapy. However, these monotherapies are associated with a high rate of recurrence. Recent articles have proposed new therapeutic approaches such as microneedles (MNs), hydrogels, and exosomes. MNs offer a non‐invasive and painless method for the sustained release of pharmaceuticals. In the context of topical drug delivery to the skin, MN systems frequently incorporate innovative materials as vehicles for drugs, thereby enhancing the therapeutic efficacy within scarred tissue [2]. Hydrogels can be formulated to incorporate a range of therapeutic agents, including organic compounds, pharmaceuticals, and bioactive substances like growth factors, genetic material, proteins/peptides, and stem cells/exosomes, to mitigate the development and progression of keloids and hypertrophic scars [3]. Adipose‐derived mesenchymal stem cell‐derived exosomes (ADSCs‐Exos) have garnered significant attention in the treatment of keloids. These exosomes are rich in bioactive molecules, including proteins, lipids, mRNAs, microRNAs, and more, playing crucial roles in intercellular communication. They can influence the proliferation, migration of keloid fibroblasts (Kfbs), and the remodeling of the extracellular matrix (ECM). ADSCs‐Exos contribute to reducing scar formation and promoting scar‐free wound healing by regulating immune responses and inflammation, promoting angiogenesis, accelerating tissue cell proliferation, and inhibiting collagen remodeling during scar proliferation [4]. However, there is no optimal treatment that can completely prevent the formation, progression, and recurrence of pathologic scars; thus, it is essential to investigate the underlying mechanism involved in keloid formation.

Despite the involvement of numerous factors, local mechanical stretch‐tension is considered to play a crucial role in the formation of keloids. Mechanical stretch‐tension is recognized for its impact on the expression of genes associated with ECM and inflammation, which play a role in scar formation. Aarabi et al. [5] were the first to demonstrate that mechanical signal transduction affects skin wound healing in vivo. Their research indicated that applying mechanical stress during the initial stages of healing can lead to hypertrophic scarring. Employing an in vitro model to examine the consequences of subjecting cells to cyclic stretching, it was also observed that elevated tension promotes the proliferation of human fibroblasts and enhances the mechanical strength of the tissue [6]. In the formation of pathological scars, the leading edge of keloids is propelled by directional mechanical forces, resulting in heightened inflammation and local collagen production at the forefront [7]. Dohi et al. [8] found that the magnitude of mechanical stress is highest in keloid tissue, followed by peripheral tissue adjacent to these scars, and then healthy skin that has not been injured. The increased strain in the surrounding tissue may be the capability driving the outward progression of keloids. That also explains the clinical observation that keloids are more frequently noted in the anterior chest, shoulder, and other anatomical sites with greater tension [9]. The decrease in local tension is responsible for the decrease in keloid formation. Human dermal fibroblast is sensitive to mechanical stretch‐tension and is the key cell during scar formation [10]. However, the mechanism by which mechanical stretch‐tension impacts Kfbs is poorly understood. Therefore, research into the key downstream protein and signaling pathway is essential.

Thrombospondin‐1 (TSP‐1) is a multifunctional ECM glycoprotein that is secreted and released by injured tissues. In recent studies, TSP‐1 has been identified as a tension‐sensitive protein, and its role in other tension‐induced diseases is gradually being revealed. In scleroderma, mechanical tension increased TSP‐1 expression in dermal fibroblasts, while blocking TSP‐1 decreased fibroblast contraction and α‐SMA expression [10]. In our previous study, we discovered that TSP‐1 is highly expressed in hypertrophic scar tissue. This finding was subsequently validated by administrating a TSP‐1 antagonist to a rat model of hypertrophic scarring, which effectively suppressed scar formation [11]. Therefore, we hypothesized that the expression of TSP‐1 induced by mechanical stretch‐tension could be a crucial mediator of keloid formation.

In this article, we introduced a simple mechanical stretching device to investigate the effects of optimal stretch on Kfbs. Then, we demonstrated the changes in TSP‐1 expression under mechanical stretch in vitro and in vivo. In addition, we examined the effects of TSP‐1 knockdown on Kfbs after mechanical stretch. Finally, we identified the possible signaling pathway that is activated in stretch‐induced keloid formation by the high expression of TSP‐1.

## 2 Materials and Methods

### 2.1 Patient Samples

Human keloid tissues were isolated from 12 patients at our Hospital; all patient data are presented in Table 1. This is conducted with the approval of the Ethics Committee of Hospital (2021‐373) and in accordance with the principles of the Helsinki Declaration. All patients provided written informed consent.

**Table 1:** The profile of each sample for primary culture.

	Sex	Age (years)	Biopsy site	Duration of the lesion (years)	Etiology	Previous treatment
KF1	Female	23	Shoulder	5	Acne	None
KF2	Female	21	Chest	2	Acne	None
KF3	Male	26	Chest	5	Acne	None
KF4	Male	29	Chest	7	Acne	None
KF5	Female	25	Shoulder	7	Acne	None
KF6	Male	25	Chest	5	Acne	None
KF7	Male	23	Shoulder	2	Acne	None
KF8	Female	20	Chest	3	Acne	None
KF9	Female	27	Chset	3	Acne	None
KF10	Female	39	Ear lobe	5	Ear piercing	None
KF11	Female	25	Ear lobe	2	Ear piercing	None
KF12	Female	25	Ear lobe	3	Ear piercing	None

Abbreviation: KF, keloid fibroblast.

### 2.2 Isolation and Culture of Human Kfbs

Human keloid tissues were cut into 2 mm × 2 mm pieces. These small fragments were then digested at 37°C for 4 h with Collagenase solution (0.2 mg/mL; Roche). After filtration, centrifugation, and resuspension, the fibroblasts were cultured in DMEM (Gibco Life Technologies, USA) supplemented with 10% fetal bovine serum (Gibco, USA) and 1% penicillin/streptomycin (Gibco Life Technologies, USA). The medium was changed every 4 days, and experiments were performed with fibroblasts between the third and eighth passages.

### 2.3 Mechanical Stretch Devices

**Figure 1:**
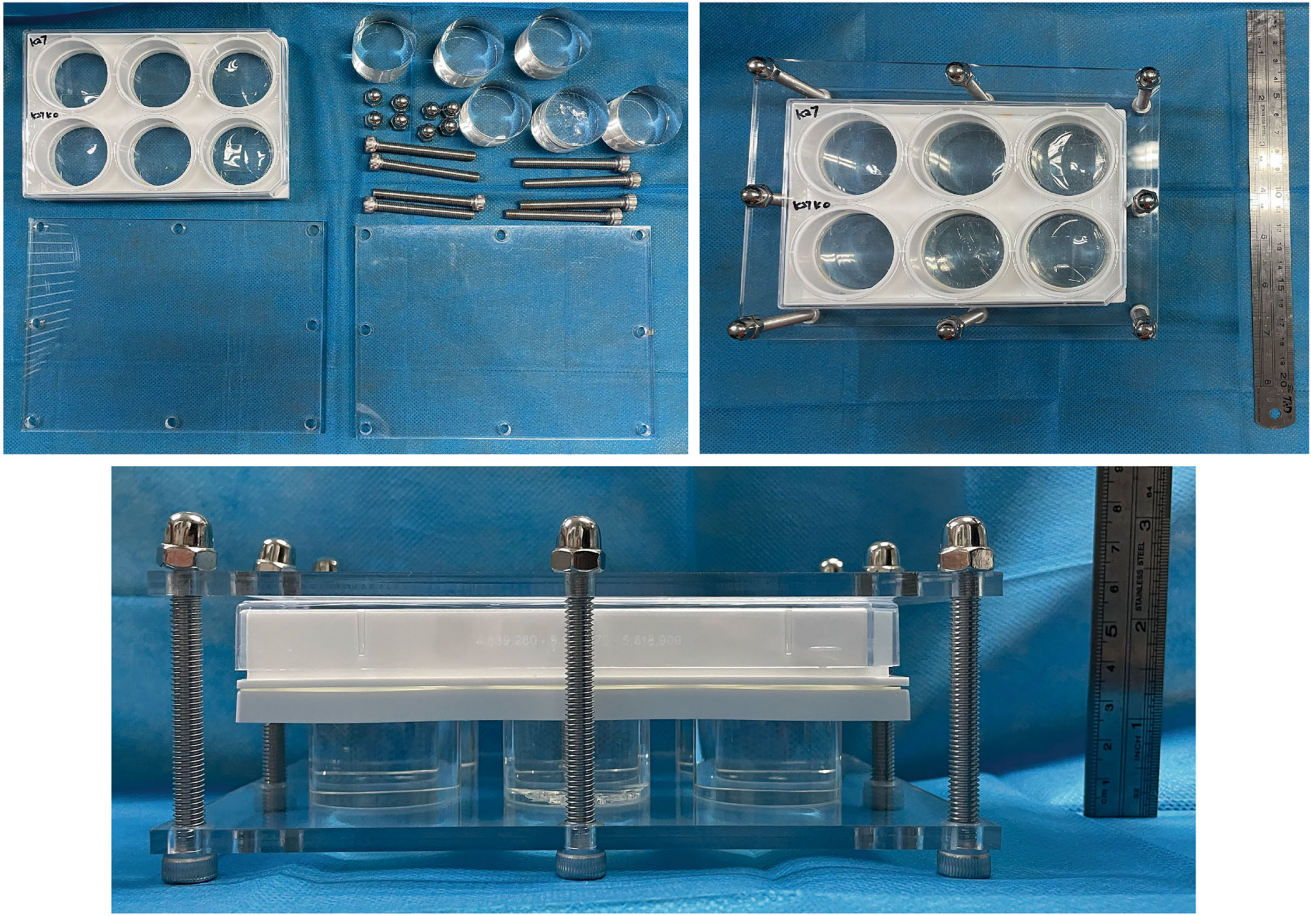
The image of a simple customized mechanical stretch device.

A simple customized mechanical stretch device was used in this study (Figure 1). The device was prepared by evenly placing six screws along the edge of the plexiglass. On the plexiglass, six polyvinyl chloride (PVC) discs were placed in accordance with six‐well flexible silicone rubber BioFlex plates (Flexcell International Corporation, Hillsborough, NC, USA). The other piece of plexiglass with six holes was used as a cover for the six‐well culture plate, and the six screws used to secure the bottom piece of plexiglass were threaded up through the holes before being capped with nuts.

### 2.4 Application of Homogeneous Equibiaxial Stretch (HES)

To prepare for the application of HES, fibroblasts were seeded at a density of 1 × 10^5^ per well in six‐well flexible silicone rubber BioFlex plates. By rotating the nuts, the upper plexiglass was pushed down, and the PVC discs caused the flexible silicone membrane of the BioFlex plate to deform and exert strain on the attached cells. The strain increased proportionally to the number of rotations of the screws, reaching a maximum of 24.08 ± 7.36% of strain after six rotations (one rotation = 360° turn of the screw).

### 2.5 Real‐Time PCR (RT‐PCR)

Total RNA was extracted using TRIzol Reagent (Invitrogen, USA) as directed in the manual. Total RNA was reverse‐transcribed into cDNA (Takara, Japan). RT‐PCR was performed with SYBR Green Taq (Takara, Japan) and the LightCycler 480 system (Roche). GADPH was used as the housekeeping gene for internal loading controls. The primers used in the study are detailed in Table 2.

**Table 2:** The primary antibody used in this study.

Gene	Direction	Sequence
TSP‐1	Forward	5′‐GGCACCAACCGCATTCCAGAG‐3′
	Reverse	5′‐ GCACAGCATCCACCAGGTCTTG‐3′
Collagen I	Forward	5′‐GAGGGCAACAGCAGGTTCACTTA‐3′
	Reverse	5′‐TCAGCACCACCGATGTCCA‐3′
Fibronectin	Forward	5′‐AGAGGCATAAGGTTCGGGAAGAGG‐3′
	Reverse	5′‐CGAGTCATCCGTAGGTTGGTTCAAG‐3′
Vimentin	Forward	5′‐CCTTCGTGAATACCAAGACCTGCTC‐3′
	Reverse	5′‐AATCCTGCTCTCCTCGCCTTCC‐3′

### 2.6 Western Blot

The fibroblasts were lysed with RIPA lysis buffer for 30 min on ice. After measuring the protein concentration using a BCA protein assay kit (Beyotime, China), equal amounts of total proteins were separated on a 10% SDS‐PAGE gel at 120 V for 1 h before being transferred to PVDF membranes. The membranes were incubated with primary antibodies against Fibronectin (Abclonal, A12932, 1:1000), Collagen I (Col I; Abcam, ab34719, 1:1000), Vimentin (Abcam, ab2413, 1:1000), TSP‐1 (Abcam, ab263905, 1:1000), and GADPH (Abcam, ab8245, 1:2000) at 4°C overnight. After 1 h of incubation with HSP secondary antibodies at room temperature, the signal was developed using the ECL system (Millipore, Billerica, MA). ImageJ was used for quantitative analysis of the relative protein concentration, which was calculated as the ratio of the target protein's density to that of GADPH (internal control).

### 2.7 shRNA and Transfection

To knock down TSP‐1, a shRNA sequence specifically targeting human TSP‐1 was cloned into the pAdTrack‐CMV plasmid. The target sequences of the TSP‐1 shRNAs were the following: 5′‐GCC AGA ACT CGG TTA CCA T‐3′. As the negative control, a scrambled sequence that did not target any known human or rat gene was used. Adenovirus was produced using the pAdEaSY‐1 system and propagated in HEK293 cells according to the manufacturer's instructions. The titer of the adenovirus was determined as 1.0 × 1010 plaque‐forming units/mL. Kfbs were then infected with an adenovirus expressing either TSP‐1 shRNA or control shRNA.

### 2.8 Cell Proliferation Assay

Cell proliferation was evaluated using the Cell Counting kit‐8 (CCK‐8; Beyotime, China) assay and the 5‐ethynyl‐2′‐deoxyuridine (EdU; Beyotime, China) assay according to the protocol. The treated fibroblasts were seeded at a density of 1 × 10^5^ per well in a 96‐well plate. After 0, 24, 48, and 72 h, 10 μL of CCK‐8 solution and 90 μL of DMEM complete medium were added to each well, followed by a 2‐h incubation at 37°C. The number of viable cells was measured at a wavelength of 450 nm. For EdU assays, treated fibroblasts were seeded at a density of 2 × 10^5^ per well in a six‐well plate. After 24 h, the treated fibroblasts were incubated with EdU for 2 h before staining. The positive cells were identified using a microscope.

### 2.9 Wound Healing Assay and Cell Migration Assay

In preparation for the wound healing assay, treated fibroblasts were seeded at a density of 2 × 10^5^ per well in a six‐well plate. The 1000 μL sterile pipette tips were used to make the scratch across the cell layer. After two washes with PBS, plates were photographed at the same location at 0, 18, and 36 h. All experiments were repeated at least three times. Cell migration assays were performed using Transwell chambers (Corning, NY, USA). Fibroblasts were suspended in serum‐free DMEM and seeded into the upper chamber at a density of 1 × 10^5^ in 200 μL medium. DMEM complete medium was added to the lower chamber and incubated for 24 h. The chambers were fixed with 4% paraformaldehyde, stained with 0.1% crystal violet (Beyotime, China), and observed through an inverted microscope. ImageJ was used to analyze five random fields at a 100× magnification on each membrane.

### 2.10 Apoptosis Assay

The TUNEL apoptosis detection kit (Beyotime, China) was used to assess cellular apoptosis according to the manual. The Cyanine‐3‐labeled TUNEL‐positive cells were imaged under a fluorescent microscope using 532 nm excitation and 588 nm emission. ImageJ was used to analyze five random fields at a magnification of 100×.

### 2.11 Animal Model

Animal modeling was performed on 8‐week‐old Sprague‐Dawley rats. All animal experiments were approved by the Animal Care and Use Committee of Peking University Shenzhen Hospital. The scar model was represented by a stretched rat tail model, as proposed by Zhou et al. [12]. First, a 6 mm × 6 mm wound was made in the rat tail. Once complete re‐epithelialization had occurred, a 2‐cm‐diameter steel rings were tied beneath the tail scar for 4 weeks to provide mechanical stretch. This study protocol complies with the ARRIVE guidelines and the humane treatment of research animals.

### 2.12 Hematoxylin‐Eosin Staining

Tissue samples were fixed with 4% paraformaldehyde after surgery. The sections were then stained with haematoxylin‐eosin staining methods.

### 2.13 Histology and Immunohistochemistry

Human and rat tail keloid tissue samples were fixed in 4% paraformaldehyde, embedded, and sectioned. After deparaffinization and rehydration, sections were stained with an anti‐TSP‐1 primary antibody (Abcam, ab267388, 1:250) and an HRP‐conjugated goat anti‐rabbit secondary antibody (Abclonal, AC028, 1:250). Mean optical density measurements were analyzed through ImageJ.

### 2.14 Statistical Analysis

A two‐tailed Student's *t*‐test was utilized for comparisons between two groups. One‐way ANOVA was used to compare multiple groups. *p* < 0.05 indicated a statistically significant difference. Each experiment consisted of three replicates. The results are expressed as means ± SD. A priori significance was set at *α* = 0.05 for all analyses. All hypothesis tests were two‐sided. Analyses were conducted using GraphPad Prism, Version 9.5.1(528).

## 3 Results

### 3.1 Attainment and Measurement of HES

**Figure 2:**
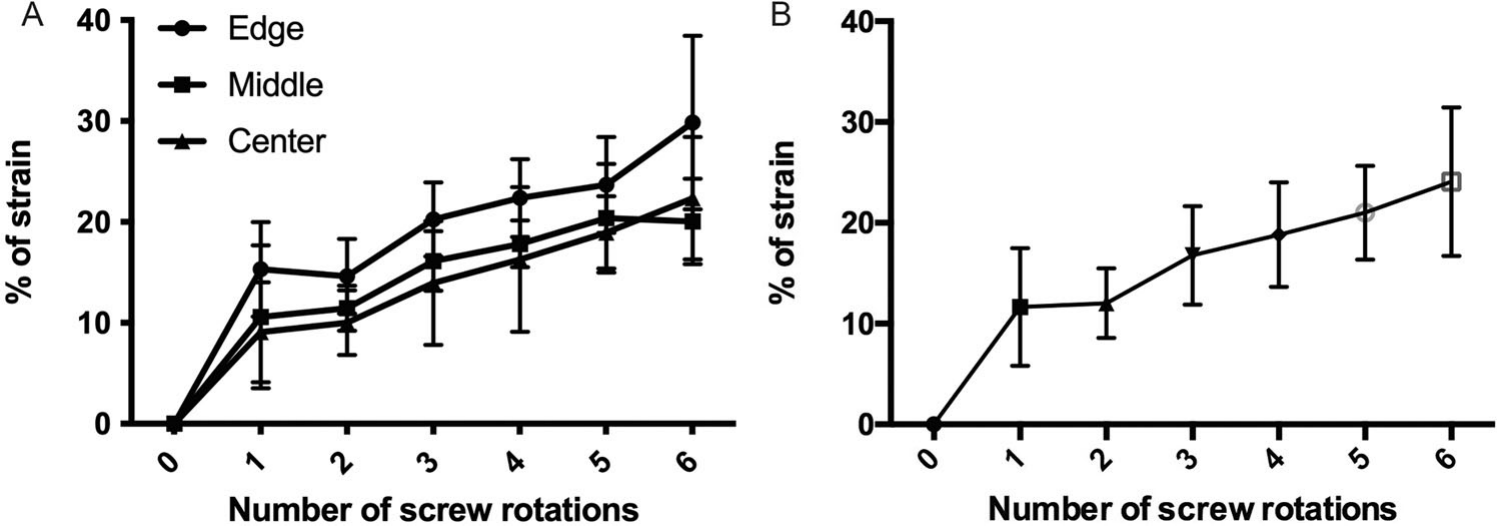
The measurement of homogeneous equibiaxial stretch (HES). The percentage of strain applied to the silicon membranes was determined microscopically by varying the number of screw rotations, which ranged from 1 to 6. (A) Following the application of HES via screw rotations, the image demonstrated the percentage of HES attained after eachscrew rotation at various points of the elastic membranes. (B) The image showed the average percentage of HES attained after each screw rotation. The measurements were repeated 10 times.

This simple self‐assembling device was designed for in vitro stretch experiments for human samples under any research conditions. Our device's method for applying HES to elastic membranes is based on the prototype described by Rana et al. [13]. Under non‐stretched conditions, 1 mm dots were marked at the center, middle, and edge of the membrane. The amount of static stretch can be varied by adjusting the number of screw rotations, which range from 1 to 6. The expansions of the dots were measured using a microscope, as proposed by Rana et al. [13]. Figure 2A demonstrates the relationship between the percentage of HES attained after each rotation of the screws at various points on the elastic membranes. To quantify the tension that HES applied to Kfbs cultured on elastic membranes, Figure 2B depicts the average percentage of HES achieved after each screw rotation.

### 3.2 Protein and mRNA Expression in Kfbs Subjected to Optimal HES (oHES)

**Figure 3:**
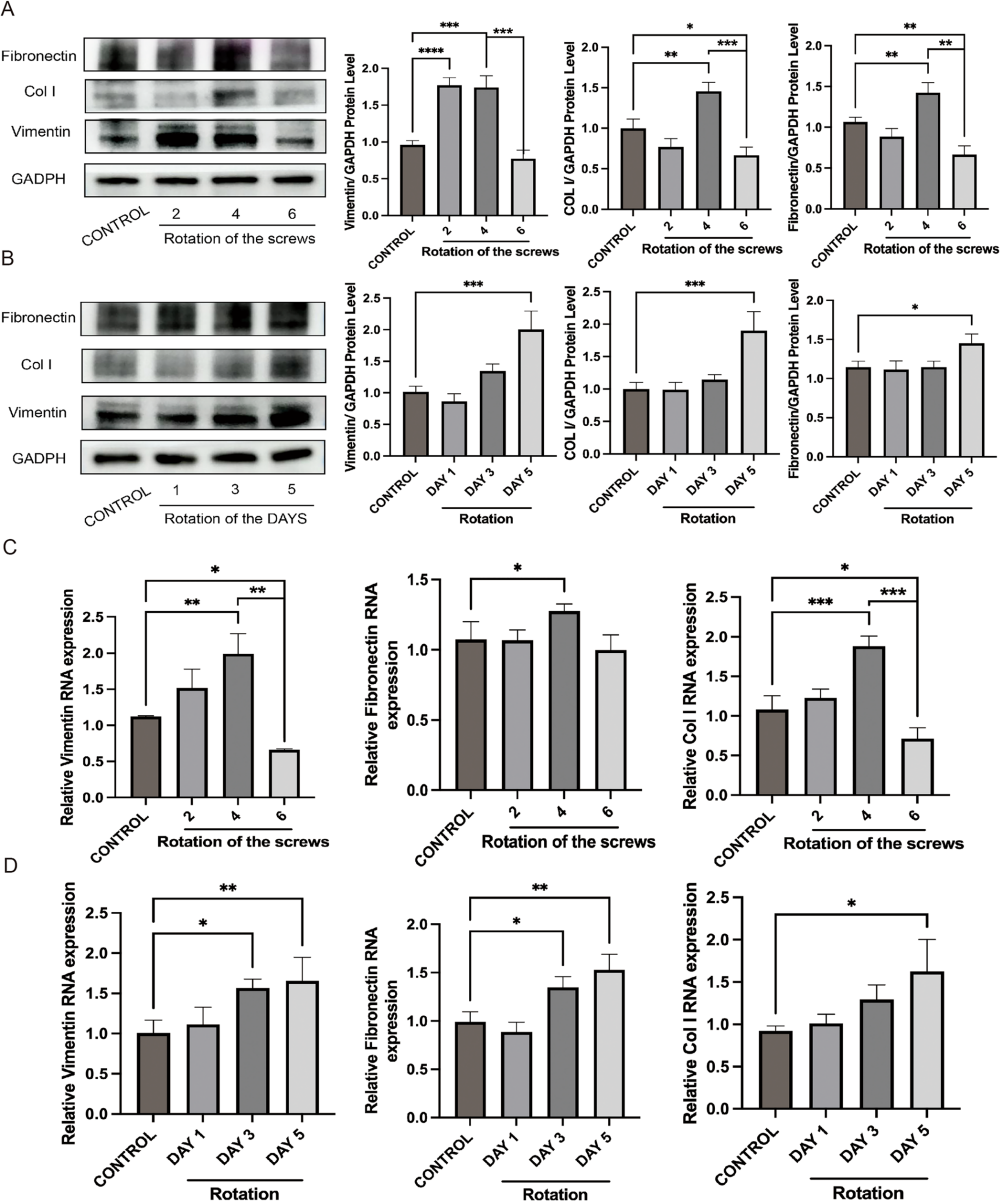
The effects of oHES on the protein and mRNA levels of Col I, vimentin, and fibronectin in Kfbs. After loading Kfbs with different stretch strengths (0, 2, 4, 6 screw rotations) for 0, 1, 3, and 5 days, the results showed that protein (A and B) and mRNA (C and D) levels of vimentin, Col I, and fibronectin were significantly higher on Day 5 when four screw rotations were applied (*p < 0.05; **p < 0.01; ***p < 0.001; ****p < 0.0001).

To examine the protein and mRNA expression in Kfbs subjected to HES, Western blot and RT‐PCR were used after Kfbs were exposed to varying degrees and durations of HES. First, the protein levels of vimentin, Col I, and fibronectin in Kfbs exposed to varying degrees and durations of stretching were examined. At four rotations of the screws, the expression of vimentin, Col I, and fibronectin was significantly higher compared to the control (Figure 3A, vimentin *p* < 0.001, Col I *p* < 0.001, and fibronectin *p* < 0.001). In addition, the protein expression of vimentin, Col I, and fibronectin was significantly increased on Day 5 when four rotations of the screws were applied (Figure 3B, vimentin *p* < 0.001, Col I *p* < 0.001, and fibronectin *p* = 0.02). Similar trends were also observed in the expression of their mRNA (Figure 3C, vimentin *p* < 0.001, Col I *p* < 0.001, and fibronectin *p* = 0.03; Figure 3D, vimentin *p* < 0.001, Col I *p* = 0.02, and fibronectin *p* < 0.001). These findings indicated that four screw rotations (18.83 ± 5.17% of strain) for 5 days could result in the attainment of oHES, leading to a significant increase in the protein and mRNA levels of vimentin, Col I, and fibronectin. Subsequent experiments were conducted under optimal experimental conditions.

### 3.3 oHES Accelerated Kfbs Proliferation and Migration But Not Apoptosis

**Figure 4:**
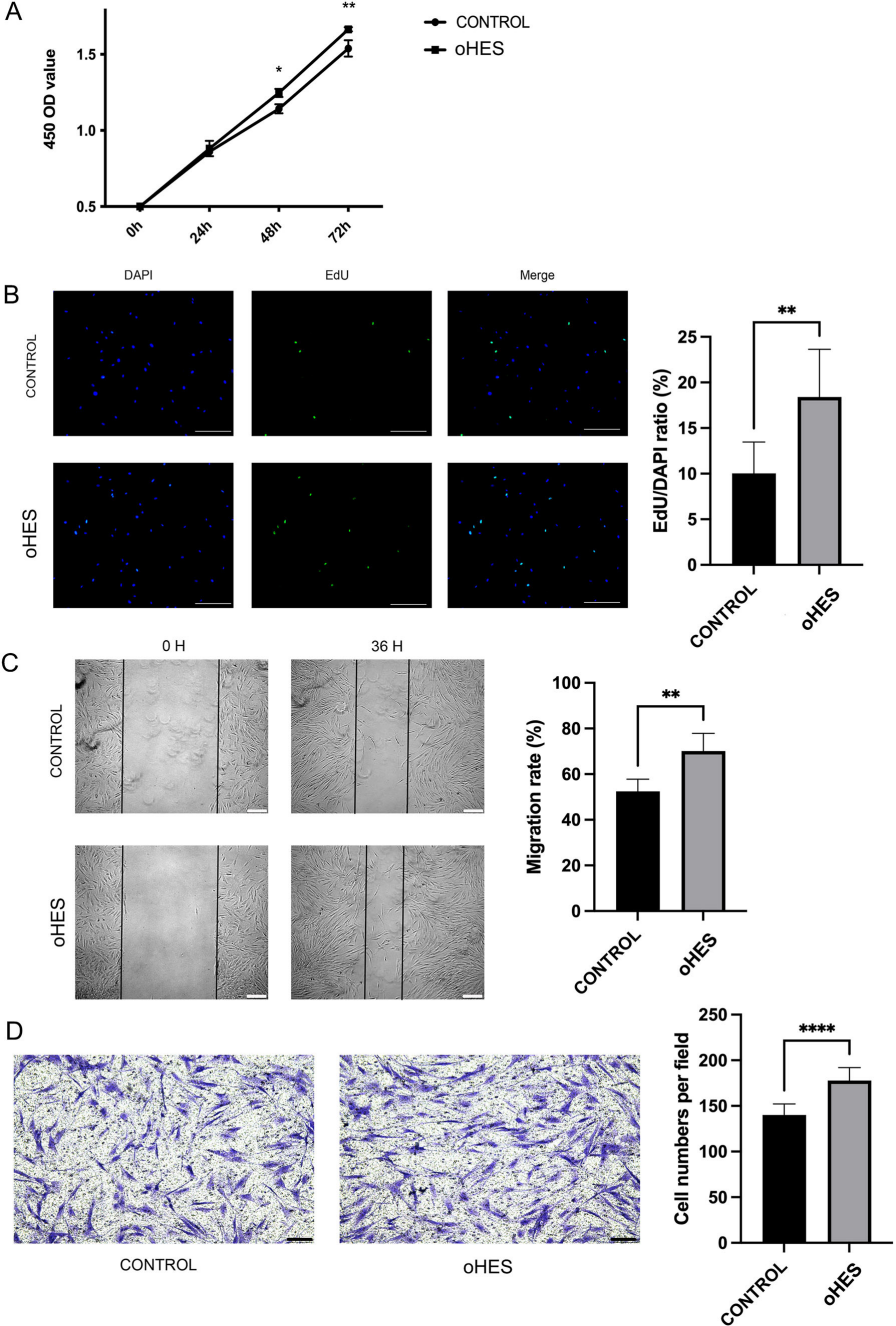
oHES promoted the proliferation and migration of Kfbs. (A) After 5 days of HES loading on the Kfbs, the cell proliferation function, as measured by CCK‐8, increased significantly, especially at 48 and 72 h. (B) The EdU assay showed that the proportion of cells in the proliferative phase increased significantly compared to the control group (Scale bar = 250 μM). (C) Upon oHES loading, the wound healing assay demonstrated that the rate of cell migration was significantly accelerated at 36 h relative to the control group. The black lines denoted the edges of the cell wound area. The migration rate was quantified as the percentage of cells migrating into the wound relative to the clear area at 0 h (Scale bar = 250 μM). (D) Transwell assay was performed on Kfbs with and without oHES loading. Both images of the Transwell assay were captured at 24 h (Scale bar = 100 μM) (*n* = 5; *p < 0.05; **p < 0.01; ****p < 0.0001).

CCK‐8 and EdU were used to evaluate the ability of oHES to induce Kfbs proliferation. Both results of CCK‐8 and EdU demonstrated that oHES loading accelerated the proliferation of Kfbs after pHES loading (Figure 4A, *p* < 0.001; Figure 4B, *p* < 0.001). Next, wound healing assay and Transwell migration assay were used to measure the motility of oHES‐induced Kfbs. In both the wound healing assay and Transwell migration assay, oHES significantly increased Kfbs migration compared to the control group(Figure 4C, *p* < 0.001; Figure 4D, *p* < 0.001). Also, cell apoptosis between the oHES groups and the control group was measured using the TUNEL assay. The results, however, showed no statistically significant differences (data were not shown). Taken together, these findings demonstrated that oHES could induce Kfbs proliferation and migration.

### 3.4 oHES Induced Expression of TSP‐1 In Vitro and In Vivo

**Figure 5:**
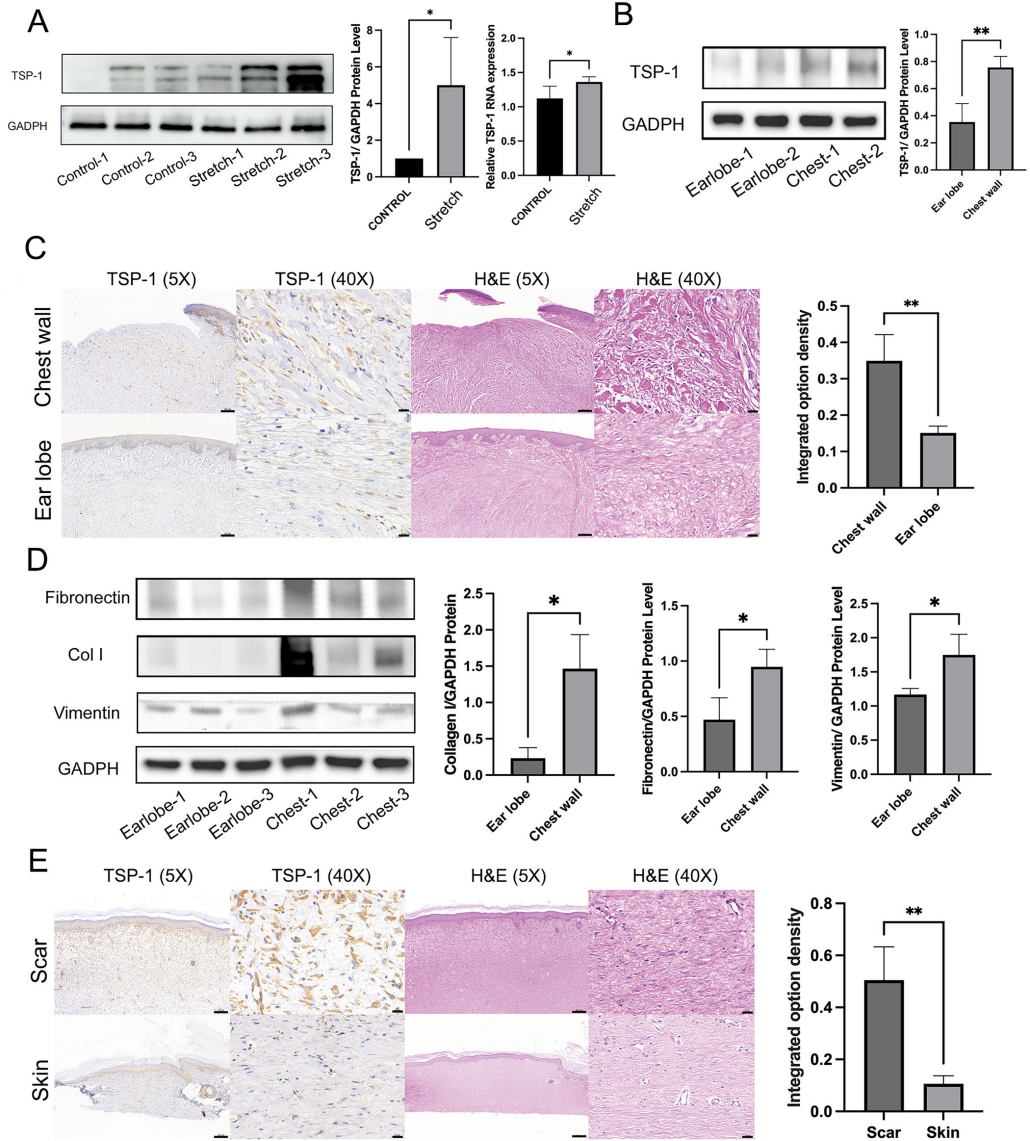
TSP‐1 levels in oHES‐loaded Kfbs in both human keloid tissue and animal models. (A) Both the protein and mRNA levels of TSP‐1 in Kfbs increased significantly after oHES loading (*n* = 3). Human keloid tissue samples from the chest wall and ear lobe represented keloid with or without oHES‐loading, respectively. (B) The protein levels of TSP‐1 significantly increased in Kfbs from chest wall (*n* = 3). (C) H&E staining showed bulky collagen fiber in keloid tissue from chest wall. IHC staining and integrated option density images revealed that keloid from chest wall highly expressed TSP‐1 protein. (D) The protein levels of Col I, vimentin, and fibronectin significantly increased in Kfbs from chest wall (*n* = 3). (E) Tension‐induced rat tail scars represented oHES‐loaded pathological scars. IHC staining and integrated option density images revealed that keloid from chest wall and pathological scars loaded with oHES highly expressed TSP‐1 protein (*p < 0.05; **p < 0.01; Scale bar, 5× = 200 μM; 40× = 20 μM). HES, homogeneous equibiaxial stretch; oHES, optimal homogeneous equibiaxial stretch.

RT‐PCR and Western blot were used to demonstrate the expression of TSP‐1 in Kfbs following application of oHES. Western blot confirmed that the protein levels of TSP‐1 were elevated in oHES‐loaded Kfbs. RT‐PCR showed that the mRNA level of TSP‐1 was significantly higher in oHES‐loaded Kfbs (Figure 5A, protein level *p* = 0.03; mRNA level *p* = 0.04). To further verify whether TSP‐1 is involved in stretch‐tension‐induced scar formation, Western blot and immunohistochemistry were used to detect and compare human keloid tissue in high stretch‐tension regions, such as the chest wall, with low stretch‐tension regions, such as the ear lobe. We discovered that regions of keloid tissues with high stretch‐tension expressed more TSP‐1 protein than those with low stretch‐tension (Figure 5B, *p* < 0.001 and Figure 5C, *p* < 0.001). Also, compared to Kfbs in the earlobe, Kfbs in the chest wall expressed higher levels of Col I, vimentin, and fibronectin at the protein levels (Figure 5D, Col I *p* = 0.02, vimentin *p* = 0.04, and fibronectin *p* = 0.03). Furthermore, to further investigate the expression of TSP1 during scar formation under tension, the study revealed that the expression level of TSP‐1 in the scar tissue of rat tails subjected to tension was significantly higher than that observed in the skin of control rat tails. This finding further corroborates the aforementioned results (Figure 5E, *p* < 0.001). Thus, these results supported the hypothesis that TSP‐1 is a key protein in stretch‐tension‐induced scar formation.

### 3.5 TSP‐1 Knockdown Inhibited Vimentin, Col I, and Fibronectin Expression, and Subsequent Cellular Functions in oHES‐Loaded Kfbs

**Figure 6:**
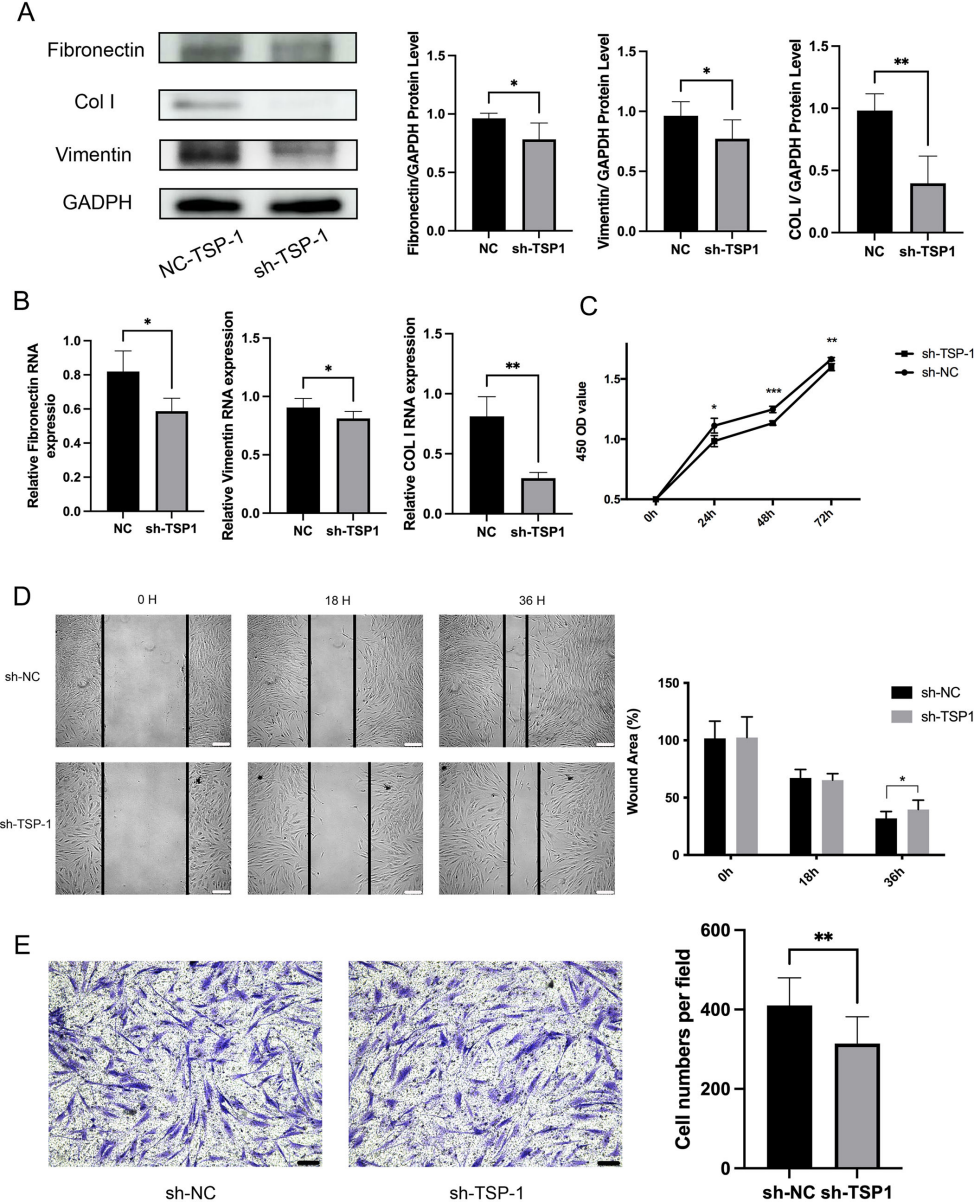
TSP‐1 knockdown in Kfbs inhibited oHES‐induced cellular functions and protein expression. After knocking down TSP‐1 by transfecting shRNA into Kfbs, the protein (A) and mRNA (B) levels of Col I, vimentin, and fibronectin were significantly decreased following oHES loading. (C) CCK‐8 assay showed a decline in cell proliferative function 24, 48, and 72 h after TSP‐1 knockdown. (D) The image of wound healing assay demonstrated that the ability of cell migration decreased significantly at 36 h compared to control group. Black lines denoted the edges of cell wound area (Scale bar = 250 μM). (E) Transwell assay was conducted on Kfbs, which were performed with and without sh‐TSP‐1. Both images of Transwell assay were captured at 24 h (Scale bar = 100 μM) (*n* = 3; *p < 0.05; **p < 0.01; ***p < 0.001).

Results between TSP‐1 expression and oHES strongly suggested that TSP‐1 may be a significant factor in oHES‐induced keloid formation. To address the functional significance of TSP‐1 in oHES‐induced biological behaviors of Kfbs, Kfbs were transduced with an adenovirus expressing TSP‐1‐targeting shRNA (sh‐TSP‐1) or a control shRNA (sh‐NC). By the Western blot and RT‐PCR, the data showed that sh‐TSP‐1 significantly reduced the protein (Figure 6A, fibronectin *p* = 0.03; vimentin *p* = 0.03; Col I *p* < 0.001) and mRNA (Figure 6B, fibronectin *p* = 0.02; vimentin *p* = 0.04; Col I *p* < 0.001) expression of vimentin, Col I, and fibronectin in oHES‐loaded Kfbs. TSP‐1 knockdown resulted in a decrease in cell proliferation (Figure 6C, *p* < 0.001) and migration (Figure 6D, *p* = 0.03, Figure 6E, *p* < 0.001) of TSP‐1 shRNA cells relative to sh‐NC cells. The results indicated that the absence of TSP‐1 expression may be the crucial factor inhibiting the protein and mRNA expression, as well as the cellular function mediated by oHES‐loaded Kfbs.

### 3.6 TSP‐1 May Inhibit oHES‐Induced Keloid Formation via the Akt and GSK‐3β/β‐Catenin Signaling Pathways

**Figure 7:**
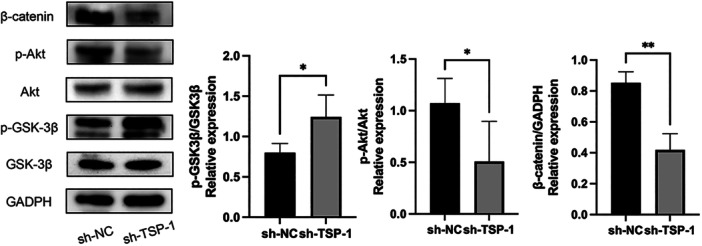
Knocking down TSP‐1 in Kfbs regulated oHES‐induced Akt and GSK‐3β/β‐catenin signaling pathways. Kfbs were transfected with sh‐NC or sh‐TSP‐1 before oHES loading to determine the effect of TSP‐1 on the signaling pathway. After oHES loading, phosphorylated Akt in sh‐TSP‐1 Kfbs decreased significantly, while phosphorylated GSK‐3β increased significantly. Total β‐catenin also decreased in Kfbs upon TSP‐1 knockdown (*p < 0.05; **p < 0.01). oHES, optimal homogeneous equibiaxial stretch.

To investigate further the mechanism of TSP‐1 expression caused by oHES during keloid formation, Kfbs were transfected with sh‐TSP‐1 or sh‐NC before loading with oHES. Protein expression of the mechanism was assessed with a Western blot. The expression levels of p‐Akt and β‐catenin were significantly decreased in oHES‐loaded sh‐TSP‐1 transfected fibroblasts (Figure 7, p‐GSK‐3β/GSK‐3β *p* = 0.03; p‐Akt/Akt *p* = 0.02; β‐catenin/GADPH *p* < 0.001). The expression of p‐GSK‐3β was significantly increased in oHES‐loaded sh‐TSP‐1‐transfected fibroblasts. Collectively, these results indicated that the exacerbating abilities enhanced by oHES in Kfbs are dependent on TSP‐1 induction through the downstream Akt and GSK‐3β/β‐catenin signaling pathways.

## 4 Discussion

Although the relationship between mechanical force and keloid formation has been extensively discussed, the underlying mechanisms remain unknown. To investigate the role of the mechanical stretch‐tension environment during keloid formation, a customized cell culture system was used to apply mechanical stretch‐tension to Kfbs. The most commonly used stretch‐tension culture devices are the Tesion Cells Stretching Bioreactor System (Flexcell) and the Strex Cell Stretching System (STREX). The advantage of these devices was that they automatically applied the specified tension to cultured cells. However, these devices are expensive and must be stored in a separate cell incubator for experiments, which most laboratories cannot afford. In this study, we used a self‐assembled mechanical stretch‐tension cell culture system, which is comparable to the device used in the previous study [13]. The highlight of this study was that our device could apply a stretch by up to 24.08 ± 7.36% more than the other self‐made devices described previously [14]. In this article, we presented a simple, cost‐effective device capable of producing strains of up to 24%.

Fibroblasts have been extensively investigated in biomechanical models. ECM in the dermis is primarily responsible for the mechanical properties of the skin. Collagen fibers are accountable for the stiffness and mechanical strength of the tissue [15]. In this article, the protein and mRNA levels of Col I, fibronectin, and vimentin in oHES‐loaded Kfbs were significantly increased (at four screw rotations, 18.83 ± 5.17% of strain, for 5 days), with Col I being the most prominent. Also, the proliferation and migration abilities of Kfbs were significantly increased after being loaded with oHES. During bronchospasm in asthmatic patients, exposure to high intensities (18%), compared to physiological strain (5%), compromised the integrity of the endothelial barrier and cell contractility [16]. Kawai et al. [17] showed that the effect of 20% stretching force on wounds increases the risk of keloid formation by involving fibroblasts. These results suggested that optimal stretch‐tension may promote keloid formation. However, this article initially suggested that excessive tension (six screw rotations stretch strength, 24.08 ± 7.36% of strain) could inhibit scar formation. Similar to clinical observations, by implanting customized expanders beneath the keloid, keloid growth was halted or even partially regressed during expansion, as previously reported by Wu et al. [18]. However, the specific molecular biological pathways remain unidentified. Altogether, oHES has to potential to enhance cell proliferative and migratory functions by increasing the expression of Col I, fibronectin, and vimentin.

TSP‐1 levels in scar fibroblasts are elevated in both hypertrophic scar and keloid [19]. Recent research has identified TSP‐1 and its receptors as tension‐sensitive proteins [20, 21]. In our previous study, LSKL, an inhibitor of TSP‐1, was utilized to reduce the formation of hypertrophic scars in a stretched rat tail scar model [11]. Previous results revealed that TSP‐1 may be involved in the formation of stretch‐induced scar. In this article, these results showed that oHES could increase the expression of TSP‐1 in Kfbs. As opposed to keloids originating from the earlobe, keloids originating from the chest wall are considered scar formations induced by a high‐tension environment. To further verify the correlation between TSP‐1 and the common sites of human keloids, we performed immunohistochemical detection on a keloid derived from the chest wall and earlobe. In addition, the rat tail scar animal model constructed by tensile stress confirmed that TSP‐1 may be a key protein in the formation of stretch‐tension‐induced keloid. Also, by knocking down TSP‐1, the expression of vimentin, fibronectin, and especially Col I (decreased by 58.59% compared to the NC group) in Kfbs could be significantly reversed in oHES‐loaded Kfbs. Numerous articles have confirmed that Col I is a key protein in keloid formation. These results suggested that in the future, a TSP‐1 targeted therapy may be more effective at inhibiting mechanical stretch‐induced keloid formation in the presence of high Col I protein expression.

In the pathological process of keloid formation, keloid cells sensed mechanical stretch changes in their microenvironment and activated the mechanical signal pathway, thereby transforming mechanical stimuli into biochemical signals and ultimately affecting cell functions [22]. Several sensitive mechanical signal pathways, such as transforming growth factor beta (TGF‐β)/Smad, Akt, Wnt, FAK, MAPK/ERK, YAP/TAZ, integrin, and calcium ion signaling pathways, have been introduced during keloid progression [23]. Aarabi et al. [5] were the first to demonstrate the impact of mechanical signal transduction on wound healing during scar formation. In a mechanically stretch‐induced rat scar model, they proved that an increase in mechanical stretch could induce hypertrophic scar formation through the Akt pathway. A previous study in our lab demonstrated that the TSP‐1 inhibitor, LSKL, could dose‐dependently inhibit the expression of Col I in scar fibroblasts through the Akt signaling pathway [11]. In this study, knocking down TSP‐1 significantly inhibited the expression of p‐Akt in oHES‐loaded Kfbs.

**Figure 8:**
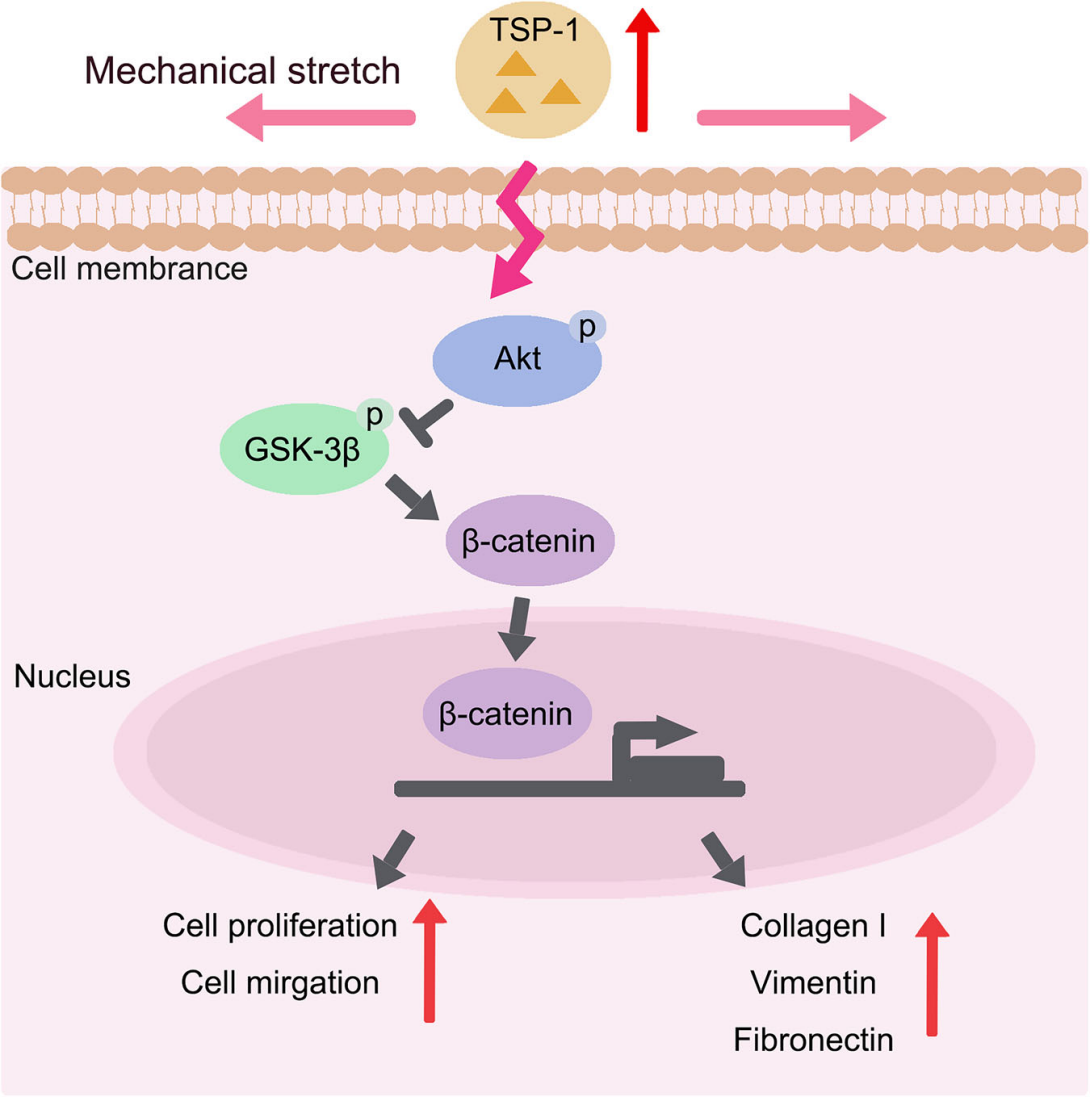
A schematic representation of how oHES promotes TSP‐1 expression through the Akt and GSK‐3β/β‐catenin signaling pathways.

To discover how the Akt network is wired and integrated with other cellular signals, the article discussed GSK‐3β, one of the key multifunctional downstream signaling nodes [24]. Cheon et al. [25] demonstrated that growth factors such as TGF‐β could elevate the level of β‐catenin by inhibiting GSK‐3β activity and prolong the proliferative phase of wound healing, resulting in a scar, during the wound healing process. Also, Igota et al. [26] found that an increase in p‐GSK‐3β and a decrease in β‐catenin in Kfbs could inhibit the keloid formation. Kfbs expressed a significant amount of β‐catenin, suggesting a role for this protein in fibroproliferative diseases [27]. Dohi et al. [8] found that the expression of β‐catenin, which was correlated with the mechanical transduction pathway and stretch, was highest in keloid tissue compared to normal skin. Notably, the AKT/GSK‐3β pathway plays a crucial role in pathological fibrotic responses [28]. TGF‐β stimulates the phosphorylation of AKT through the activation of phosphoinositide 3‐kinase. This activated AKT then directly phosphorylates and inactivates GSK3‐β, which is a critical modulator of the overall protein translation machinery [29]. As a result, in this study, knocking down TSP‐1 in oHES‐loaded Kfbs significantly inhibited the expression of Col I by increasing p‐GSK‐3β levels and decreasing β‐catenin levels. Lee et al. [30] also confirmed their findings using a Wnt receptor decoy. These results suggested that mechanical stretch‐tension may promote keloid scar formation through the Akt and GSK‐3β/β‐catenin signaling pathway (Figure 8).

Nonetheless, this article contains some limitations. First, this simple cell tension device was incapable of accurately reflecting the true mechanical stretch received by a human scar. Second, the device was unable to provide the specified newtons of force. The field will make significant strides forward in the future if researchers are able to create precise devices that can adequately simulate the human mechanical microenvironment.

In conclusion, these results confirmed that an oHES can enhance the proliferative and migratory functions of Kfbs by increasing the expression of Col I, fibronectin, and vimentin. In addition, the high expression of TSP‐1 in Kfbs after stretching, which was observed in human keloid tissue derived from the chest wall and in a stretch‐induced rat scar model, indicated that TSP‐1 may be a crucial mediator of oHES‐induced keloid formation. Inhibiting TSP‐1 with shRNA significantly reversed cell functions, protein and mRNA expression, particularly of Col I, induced by oHES. Regarding signaling pathways, inhibiting TSP‐1 may attenuate oHES‐induced keloid formation through the Akt and GSK‐3β/β‐catenin signaling pathways. Overall, these findings suggested that an optimal mechanical stretch promotes keloid formation by increasing Col I expression through the TSP‐1‐mediated Akt and GSK‐3β/β‐catenin signaling pathways.

## Author Contributions

**Xiangwen Xu:** data curation, writing – original draft, writing – review and editing. **Yihan Zhang:** data curation, writing – original draft. **Yanting Ou:** data curation. **Yixing Kang:** formal analysis. **Mengfan Wu:** methodology. **Jun Feng:** methodology. **Yun Long:** supervision. **Yongyan Cui:** supervision. **Dandan Liu:** conceptualization, investigation, project administration, writing – original draft. **Lin Luo:** conceptualization, project administration, supervision, writing – review and editing.

## Conflicts of Interest

The authors declare no conflicts of interest.

## Transparency Statement

The lead author, Lin Luo, affirms that this manuscript is an honest, accurate, and transparent account of the study being reported; that no important aspects of the study have been omitted; and that any discrepancies from the study as planned (and, if relevant, registered) have been explained.

## Data Availability

The authors confirm that the data supporting the findings of this study are available within the article [and/or] its supporting materials.
